# Fluorescence Correlation Spectroscopy Analysis of Effect of Molecular Crowding on Self-Assembly of *β*-Annulus Peptide into Artificial Viral Capsid

**DOI:** 10.3390/ijms22094754

**Published:** 2021-04-30

**Authors:** Risako Kobayashi, Hiroshi Inaba, Kazunori Matsuura

**Affiliations:** 1 Department of Chemistry and Biotechnology, Graduate School of Engineering, Tottori University, Tottori 680-8552, Japan; xiaolinlishazi86@gmail.com (R.K.); hinaba@tottori-u.ac.jp (H.I.); 2 Centre for Research on Green Sustainable Chemistry, Tottori University, Tottori 680-8552, Japan

**Keywords:** self-assembly, *β*-annulus peptide, artificial viral capsid, molecular crowding, fluorescence correlation spectroscopy

## Abstract

Recent progress in the *de novo* design of self-assembling peptides has enabled the construction of peptide-based viral capsids. Previously, we demonstrated that 24-mer *β*-annulus peptides from tomato bushy stunt virus spontaneously self-assemble into an artificial viral capsid. Here we propose to use the artificial viral capsid through the self-assembly of *β*-annulus peptide as a simple model to analyze the effect of molecular crowding environment on the formation process of viral capsid. Artificial viral capsids formed by co-assembly of fluorescent-labelled and unmodified *β*-annulus peptides in dilute aqueous solutions and under molecular crowding conditions were analyzed using fluorescence correlation spectroscopy (FCS). The apparent particle size and the dissociation constant (*K_d_)* of the assemblies decreased with increasing concentration of the molecular crowding agent, i.e., polyethylene glycol (PEG). This is the first successful in situ analysis of self-assembling process of artificial viral capsid under molecular crowding conditions.

## 1. Introduction

The interior of living cells is often referred to as the ‘molecular crowding environment’, in which many biomacromolecules, such as proteins, nucleic acids, and carbohydrates, exist in high density [1]. Over the past two decades, it has been revealed that the structure, activity, and stability of biomacromolecules in molecular crowding environments, which mimic the cellular environment, considerably differ from those in dilute aqueous solutions. Sugimoto and co-workers demonstrated that DNA duplexes were destabilized, whereas quadruplexes were stabilized in the presence of molecular crowding agents such as polyethylene glycol (PEG) [2]. It has also been reported that the excluded volume effect of molecular crowding agents significantly affected the folding structures and stability of proteins [3,4,5,6,7,8].

Spherical viral capsids, i.e., outer protein shells surrounding viral nucleic acids, are 20–100 nm nanocapsules self-assembled from their subunit proteins. In recent decades, natural spherical viral capsids have attracted considerable attention for application as nanoreactors, vaccine platforms, and nanocarriers for drug delivery systems [9,10,11,12]. Physicochemical analysis of the self-assembly processes of viral capsids is an important aspect in molecular biology and valuable for the molecular design of viral capsids as functional materials [13,14]. To elucidate the self-assembling process of natural viral capsids, intermediate structures have been previously detected by ion mobility separation-mass spectrometry, interferometric scattering microscopy, transmission electron microscopy (TEM), and atomic force microscopy (AFM) under dilute conditions [14,15,16]. Nonetheless, the formation of viral capsids by self-assembly actually occurs in molecular crowding environments, such as a host cell [17]. Mateu and co-workers reported that macromolecular crowding led to the reversible assembly of CA, human immunodeficiency virus type 1 capsid protein, into capsid-like particles at low ionic strength, which was different from diluted conditions [18]. However, with the exception of the above pioneering example, the self-assembly process of viral capsids under molecular crowding conditions is not fully understood.

Recent progress in the *de novo* design of self-assembling peptides has enabled the construction of peptide-based viral capsids [19,20,21,22,23]. We previously demonstrated that 24-mer *β*-annulus peptides (INHVGGTGGAIMAPVAVTRQLVGS), which participated in the formation of the dodecahedral internal skeleton of the tomato bushy stunt virus, spontaneously self-assembled into a hollow peptide nanocapsule (artificial viral capsid) with a size range of 30–50 nm in water [23,24]. This artificial viral capsid could encapsulate various guest molecules in its cationic interior [25,26,27]. Moreover, its exterior surface could be decorated with different functional molecules by modifying the C-terminal of the *β*-annulus peptide, which was directed toward the capsid exterior [28,29,30,31,32,33].

In this work, we propose the application of artificial viral capsids formed by the self-assembly of *β*-annulus peptides as simple models for analyzing the effects of molecular crowding environments on the process of viral capsid formation. Analyzing the self-assembly using conventional dynamic light scattering (DLS) and TEM techniques under molecular crowding conditions is difficult owing to high concentrations of crowding agents. Hence, we used fluorescence correlation spectroscopy (FCS) to selectively measure spontaneous fluorescence intensity fluctuations in a microscopic detection volume of approximately 10 fL to estimate the diffusion time and size of fluorescence molecules [34,35]. We report in situ FCS analysis of artificial viral capsids formed by the co-assembly of fluorescent BODIPY-labelled and unmodified *β*-annulus peptides in dilute aqueous solutions and under molecular crowding conditions.

## 2. Results

### 2.1. FCS Analyses of Artificial Viral Capsid Formation in Diluted Aqueous Solution

To analyze the formation of artificial viral capsids by FCS, we designed a BODIPY-labelled *β*-annulus peptide on the N-terminal side oriented inside the capsid [25]. A 25-mer *β*-annulus peptide bearing a cysteine (Cys) residue at the N-terminal (CINHVGGTGGAIMAPVAVTRQLVGS) was synthesized by standard solid-phase Fmoc chemistry. The peptide was purified by reversed-phase high-performance liquid chromatography (HPLC), and its structure was confirmed by matrix assisted laser desorption ionization-time of flight mass spectrometry (MALDI-TOF-MS) (*m/z* = 2408 [M]^+^, Figure S1). The BODIPY-*β*-annulus peptide was synthesized by a Michael addition reaction of BODIPY-maleimide with the thiol moiety of the Cys residue, purified by reversed-phase HPLC, and confirmed by MALDI-TOF-MS (*m/z* = 2822 [M]^+^, Figure S2).

**Figure 1:**
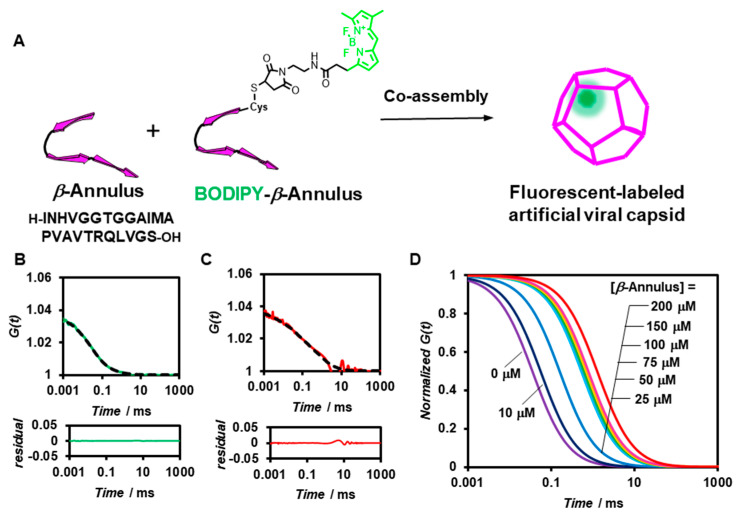
(**A**) Schematic illustration of the construction of a fluorescent-labelled artificial viral capsid by co-assembly of BODIPY-*β*-annulus and *β*-annulus peptides. (**B**,**C**) Measured (solid) and fitted (dot) autocorrelation curves for a 0.1 μM BODIPY-*β*-annulus peptide (green) and a mixture of 0.1 μM BODIPY-*β*-annulus peptide and 200 μM *β*-annulus peptide (red) measured by FCS in 10 mM Tris-HCl buffer (pH 7.0) at 25 °C. The lower graph shows the residual plot. (**D**) Normalized autocorrelation curves of a mixture of 0.1 μM BODIPY-*β*-annulus and *β*-annulus peptides at 0~200 μM in 10 mM Tris-HCl buffer (pH 7.0) at 25 °C.

The formation of artificial viral capsids by co-assembly of BODIPY-*β*-annulus and unmodified *β*-annulus peptides in a dilute aqueous solution (10 mM Tris-HCl buffer, pH 7.0) was measured by FCS (Figure 1A). The fluorescent-labelled artificial viral capsids were expected to exhibit slower autocorrelation function decay due to the larger apparent particle size than the free fluorescent-labelled peptides. Solutions of 0.1 μM BODIPY-*β*-annulus peptide and various concentrations of *β*-annulus peptides in 10 mM Tris-HCl buffer (pH 7.0) were sonicated for 10 min and incubated for 30 min at 25 °C prior to FCS analysis at 25 °C. The autocorrelation function *G*(*t*) of the aqueous solution of 0.1 μM BODIPY-*β*-annulus peptide alone showed a simple sigmoidal decay curve (Figure 1B), which fitted well with the single-component model (Materials and Methods, Equation (1)). In addition, the diffusion time was estimated to be 0.0356 ms. In the case of co-assembly of 0.1 μM BODIPY-*β*-annulus and 25~200 μM *β*-annulus peptides, *G*(*t*) was more consistent with the dual-component model (Materials and Methods, Equation (2)).

Based on the results of curve fitting at [*β*-annulus] = 200 μM, we estimated the diffusion time and ratio for the fast component to be 0.0310 ms and 38%, respectively. For the slow component the same parameters were 1.36 ms and 62%, correspondingly (Figure 1C). This suggested the coexistence of free BODIPY-*β*-annulus peptides and artificial viral capsids containing BODIPY-*β*-annulus peptides. Figure 1D illustrates the normalized autocorrelation function curves derived from the BODIPY-*β*-annulus peptide in the presence of various concentrations of the *β*-annulus peptide. The autocorrelation function exhibited a single-component curve at [*β*-annulus] = 0~10 μM and a two-step decay curve at 25~200 μM, indicating the coexistence of fast and slow components. These outcomes implied that the BODIPY-*β*-annulus peptides were incorporated into the artificial viral capsid at concentrations above 25 µM, which corresponded to the critical aggregation concentration (CAC) of the *β*-annulus peptide [24]. In other words, we confirmed that the formation of BODIPY-labelled artificial viral capsids co-assembled from BODIPY-*β*-annulus and unmodified peptides could be analyzed by FCS.

**Figure 2:**
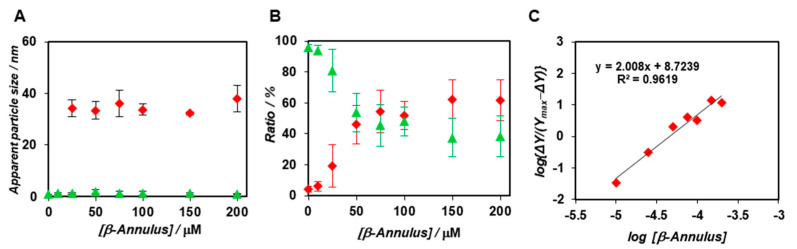
(**A**,**B**) Concentration dependence of *β*-annulus peptides on the apparent diameter (**A**) and ratio (**B**) of the fast component (green) to the slow component (red) determined by FCS curve fitting (*N* = 3) at a constant concentration of the BODIPY-*β*-annulus peptide (0.1 μM in 10 mM Tris-HCl buffer) (pH 7.0) at 25 °C. (**C**) Hill plot of binding of the *β*-annulus peptides to the BODIPY-*β*-annulus peptides in a 10 mM Tris-HCl buffer (pH 7.0). The fractions obtained from FCS analysis were fitted into the Hill equation to determine the apparent dissociation constant *K_d_*.

The apparent hydrodynamic diameter of BODIPY-labelled capsids was calculated by the Stokes–Einstein equation (Materials and Methods, Equation (4)). Moreover, the diffusion time of the BODIPY-*β*-annulus peptide was determined by FCS curve fitting (Figure S3) and plotted against the concentration of the *β*-annulus peptide (Figure 2A). The apparent particle size of the fast component was estimated to be 1 nm, which corresponded to the size of the BODIPY-*β*-annulus peptide. In contrast, the apparent particle size of the slow component appearing at concentrations above the CAC of the *β*-annulus peptide was estimated to be 30~40 nm, which was consistent with the diameter of the artificial viral capsid. These results further supported the formation of artificial viral capsids incorporating the BODIPY-*β*-annulus peptide. As the concentration of the *β*-annulus peptide increased, the ratio of the slow component increased sigmoidally (Figure 2B), indicating the formation of a cooperative assembly. The Hill equation (Materials and Methods, Equation (5)) was used to determine the dissociation constant (*K_d_* = 45.2 ± 10.6) and Hill coefficient (*n* = 2.01 ± 0.84) based on the concentration dependence (Figure 2C). 

### 2.2. FCS Analyses of Artificial Viral Capsid Formation under Molecular Crowding Conditions.

**Figure 3:**
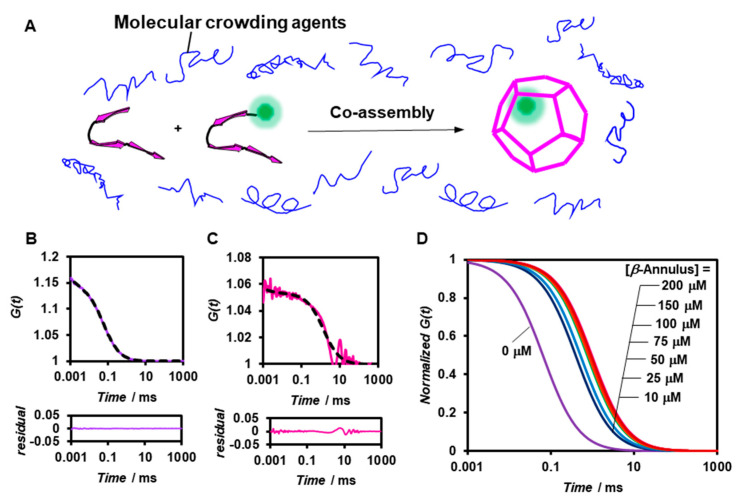
(**A**) Schematic illustration of the construction of fluorescent-labelled artificial viral capsids by co-assembly of BODIPY-*β*-annulus and *β*-annulus peptides in the presence of molecular crowding agents. (**B**,**C**) Measured (solid) and fitted (dot) autocorrelation curves for 0.1 μM BODIPY-*β*-annulus peptides (purple) and a mixture of 0.1 μM BODIPY-*β*-annulus and 200 μM *β*-annulus peptides (pink) measured by FCS in the presence of 5 wt% polyethylene glycol (PEG_2000_) in a 10 mM Tris-HCl buffer (pH 7.0) at 25 °C. The bottom graph shows residual plots. (**D**) Normalized autocorrelation curves of a mixture of 0.1 μM BODIPY-*β*-annulus and *β*-annulus peptides at 0–200 μM in the presence of 5 wt% PEG_2000_ in a 10 mM Tris-HCl buffer (pH 7.0) at 25 °C.

We subsequently analyzed the formation of artificial viral capsids under molecular crowding conditions by FCS (Figure 3A). PEG_2000_ was employed as the crowding agent and was expected to afford only the excluded volume effect without directly interacting with the *β*-annulus peptide. The viscosity of the PEG_2000_ aqueous solutions was estimated by measuring the diffusion time of the standard, namely Alexa 488 (Table S1). The *G*(*t*) of 0.1 μM BODIPY-*β*-annulus peptide alone in 5 wt% PEG_2000_-containing a 10 mM Tris-HCl buffer exhibited a simple sigmoidal decay curve (Figure 3B), which could be attributed to the presence of free BODIPY-*β*-annulus peptides. However, in the co-assembly of 0.1 μM BODIPY-*β*-annulus and 200 μM *β*-annulus peptides, the fast component with the diffusion time of 0.0230 ms (24%) was attributed to the free BODIPY-*β*-annulus peptides. In addition, the slow component with the diffusion time of 1.33 ms (76%) was ascribed to the artificial viral capsid (Figure 3C). The autocorrelation function curve derived from the BODIPY-*β*-annulus peptide in 5 wt% PEG_2000_ demonstrated that the artificial viral capsid formed at concentrations above 10 μM (Figure 3D). Intriguingly, the artificial viral capsid was formed in a 5 wt% PEG_2000_ solution at concentrations lower than the CAC (25 μM) of the *β*-annulus peptide in a dilute aqueous solution.

**Figure 4:**
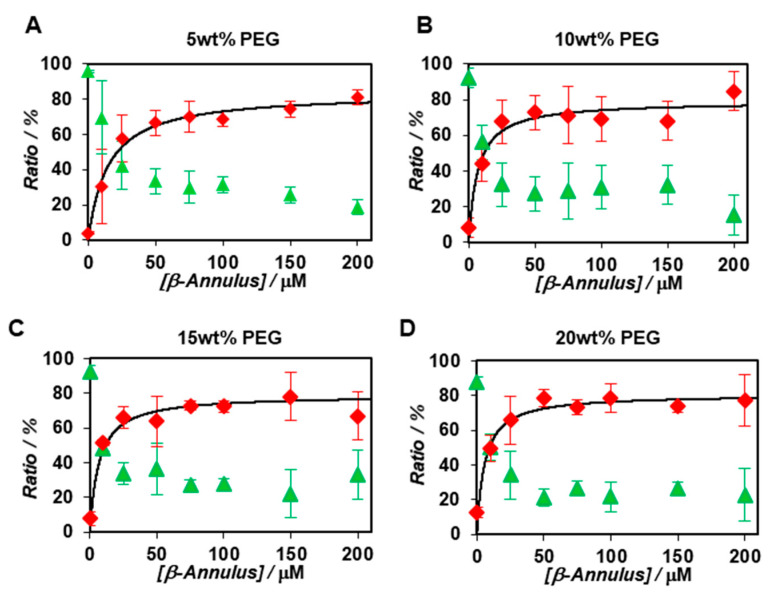
Concentration dependence of *β*-annulus peptides on the ratio of the fast (green) and slow components (red) determined by FCS curve fitting (*N* = 3) at a constant concentration of the BODIPY-*β*-annulus peptide (0.1 μM) in the presence of 5 wt % (**A**), 10 wt% (**B**), 15 wt% (**C**), 20 wt% (**D**) PEG_2000_ at 25 °C.

The apparent particle size and ratio of the co-assembly of BODIPY-*β*-annulus and *β*-annulus peptides in the presence of 5, 10, 15, and 20 wt% PEG_2000_ were examined by FCS (Figures S4–S7). Since the concentration of PEG_2000_ had a negligible effect on the diffusion time of the BODIPY-*β*-annulus peptide, there appeared to be nearly no interactions between PEG_2000_ and the peptide (Table S2). The apparent particle size of the slow component in a 5 wt% PEG_2000_ solution was approximately 30 nm, which corresponded to the diameter of the artificial virus capsid. Furthermore, the apparent particle size was approximately 10–20 nm in a 10 wt% PEG_2000_ solution and about 2–8 nm in 15 and 20 wt% PEG_2000_ solutions (Figure S8). Figure 4 shows the concentration dependence of *β*-annulus peptides on the ratio of the fast components determined by FCS curve fitting in 5~20 wt% PEG_2000_ solutions. Compared with dilute solutions (Figure 2B), the ratio of the slow component in the PEG_2000_ solution increased even at *β*-annulus peptide concentration of 10 μM (Figure 4). These results implied that the formation of the slow component was promoted in the presence of PEG_2000_. We also calculated the dissociation constants (*K_d_*) and Gibbs free energy changes (Δ*G*) in the presence of PEG_2000_ from the concentration dependence of the ratio of the slow components (Table 1).

## 3. Discussion

**Figure 5:**
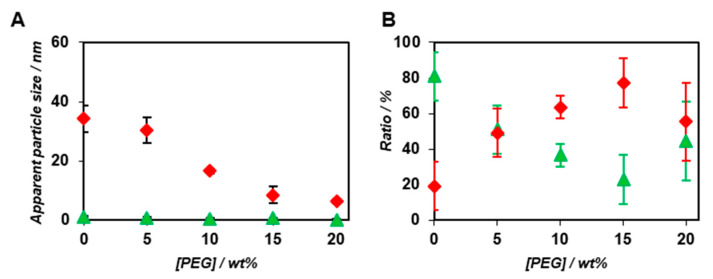
Effect of PEG_2000_ on the apparent diameter (**A**) and ratio (**B**) of the fast (green) and slow components (red) determined by FCS curve fitting (*N* = 3) in the presence of PEG_2000_ at constant concentrations of the BODIPY-*β*-annulus (0.1 μM) and *β*-annulus peptides (25 μM) at 25 °C.

Interestingly, both *K_d_* and Δ*G* decreased with the increasing concentration of PEG_2000_ (Table 1). We speculated that the exclusion volume effect of PEG_2000_ promoted the co-assembly of BODIPY-*β*-annulus and *β*-annulus peptides, which were attributed to the slow component. Figure 5 demonstrates the PEG_2000_ concentration dependence of the apparent particle size and ratio of the fast and slow components in the case of the co-assembly of 0.1 μM BODIPY-*β*-annulus peptide and 25 μM *β*-annulus peptide. The ratio of the slow components increased depending on the PEG concentration (Figure 5B). The apparent particle size of the slow component decreased with increasing PEG_2000_ concentration (Figure 5A), which implied that the complete formation of the capsid might not take place at high PEG_2000_ concentrations. The unexpected assembly at high PEG_2000_ concentrations could be attributed to the compressed capsid or micelle structures, in which the water molecules inside the capsid were excluded. Alternatively, we hypothesized that the excluded volume effect caused by high PEG_2000_ concentrations might promote the formation of capsid intermediates, e.g., a trimer of a *β*-annulus peptide with the size of approximately 3–5 nm. However, it might inhibit the growth of the entire capsid due to the high viscosity of PEG_2000_. In other words, the associative reaction promoted by the excluded volume effect of PEG_2000_ possibly involved the formation of intermediates. Hence, we expected the following effects of PEG_2000_ on the formation of the artificial viral capsids at each PEG concentration: at 5 wt% PEG_2000_, the exclusion volume effect of PEG promoted the formation of the capsid; at 10 wt% PEG_2000_, the generation of a smaller capsid was attributed to the exclusion volume effect and the viscosity of PEG; at 15 and 20 wt% PEG_2000_, the exclusion volume effect of PEG promoted the formation of intermediates; however, inhibited the generation of a capsid, which was ascribed to high viscosity of the crowding agent.

In this study, we conducted FCS analyses of the formation of fluorescent-labelled artificial viral capsids co-assembled from *β*-annulus and BODIPY-*β*-annulus peptides in dilute aqueous solutions and under molecular crowding conditions. FCS was used to selectively observe the self-assembly of *β*-annulus peptides even in the presence of molecular crowding agents. FCS evaluation of the co-assembly in a dilute aqueous solution demonstrated that the slow component with the apparent size of approximately 30 nm was formed with a dissociation constant of 45.2 μM. The dissociation constant decreased with increasing PEG_2000_ concentration, which was attributed to the exclusion volume effect. At high PEG concentrations, the size of the slow component was smaller than that of the completely formed capsid. We claim that this is the first successful in situ analysis of self-assembling process of artificial viral capsid under molecular crowding conditions.

In the near future, we plan to investigate the formation of artificial viral capsids in the presence of various other crowding agents or cell lysates. The outcomes obtained herein could provide a valuable platform for the elucidation of mechanisms of both artificial and natural viral capsid formation.

## 4. Materials and Methods

### 4.1. General

Ultrapure water of high resistivity (>18 MΩ cm) was purified using a Millipore Purification System (Milli-Q water) and was used as a solvent for the peptides. Reversed-phase HPLC was performed at ambient temperature using Shimadzu LC-6AD liquid chromatography system equipped with a UV/vis detector (220 nm, Shimadzu SPD-10AVvp, Kyoto, Japan) and Inertsil WP300 C18 column (250 × 4.6 mm and 250 × 20 mm, GL Science, Tokyo, Japan). MALDI-TOF mass spectra were obtained using an Autoflex-T2 instrument (Bruker Daltonics, Billerica, MA, USA) in linear/positive mode with α-cyano-4-hydroxy cinnamic acid (α-CHCA) as a matrix. Reagents were obtained from a commercial source and used without further purification. FCS analyses carried out on the FCS Compact BL (Hamamatsu Photonics KK, Hamamatsu, Japan) in microwell slide using a 473-nm laser.

### 4.2. Synthesis of Cys-β-Annulus Peptide.

The peptide H-Cys(Trt)-Ile-Asn(Trt)-His(Trt)-Val-Gly-Gly-Thr(tBu)-Gly-Gly-Ala-Ile-Met-Ala-Pro-Val-Ala-Val-Thr(tBu)-Arg(Pbf)-Gln(Trt)-Leu-Val-Gly-Ser(tBu)-Alko-PEG resin was synthesized on Fmoc-Ser(tBu)-Alko-PEG resin (417 mg, 0.24 mmol/g; Watanabe Chemical Ind. Ltd., Hiroshima, Japan) using Fmoc-based coupling reactions (4 equiv of Fmoc amino acid). N-Methylpyrrolidone (NMP) solution containing (1-cyano-2-ethoxy-2-oxoethylidenaminooxy) dimethylamino-morpholino-carbenium hexafluorophosphate (COMU) and diisopropylamine was used as the coupling reagent. Fmoc deprotection was achieved using 20% piperidine in *N*,*N*-dimethylformamide (DMF). Progression of the coupling reaction and Fmoc deprotection was confirmed by TNBS and chloranil test kit (Tokyo Chemical Industry Co., Ltd., Tokyo, Japan). Peptidyl-resins were washed with NMP and then dried under vacuum. Peptides were deprotected and cleaved from the resin by treatment with a cocktail of trifluoroacetic acid (TFA)/1,2-ethanedithiol/triisopropylsilane/water = 3.76/0.1/0.04/0.1 (mL) at room temperature for 3 h. Reaction mixtures were filtered to remove resins, and filtrates were concentrated in vacuo. The peptide was precipitated by adding methyl tert-butyl ether (MTBE) to the residue and the supernatant was decanted. After three times of repetitive washing with MTBE, precipitated peptide was dried in vacuo. The crude product was purified by reverse-phase HPLC eluting with a linear gradient of CH_3_CN/water containing 0.1% TFA (5/95 to 100/0 over 100 min). The fraction containing the desired peptide was lyophilized to give 1.5 mg of a flocculent solid (10.4% yield). MALDI-TOF MS (matrix: α-CHCA): *m/z* = 2410 [M + 2]^+^. The *β*-annulus peptide, which is a 24-residue peptide including without Cys, was also synthesized by the same method. 

### 4.3. Synthesis of BODIPY-β-Annulus Peptide

The obtained Cys-β-annulus peptide powder was dissolved in 20 mM sodium phosphate buffer (pH 7.0) (441.6 μL). To the solution, 3.5 mM tris(2-carboxyethyl)phosphine hydrochloride (TCEP-HCl, FUJIFILM Wako Pure Chemical Co., Osaka, Japan) in water (29.0 μL) and 17 mM BODIPY-maleimide(Funakoshi Co., Ltd., Tokyo, Japan) in dimethyl sulfoxide (29.4 μL) were added, and the mixture was incubated in the dark at 25 °C for 12 h (final concentration: 500 μM Cys-β-annulus peptide, 1 mM TCEP-HCl, 1 mM BODIPY-maleimide). After dialysis (Spectra/por7, cutoff Mw 1000, Spectrum Laboratories, Inc.,Rancho Dominguez, CA, USA) in water for 24 h, the sample was purified by reverse-phase HPLC eluting with a linear gradient of CH_3_CN/water containing 0.1% TFA (5/95 to 100/0 over 100 min). The fraction containing the desired peptide was lyophilized and dissolved in water (100 μL) to give an aqueous solution of 47.3 μM BODIPY-*β*-annulus (18.9% yield). MALDI-TOF MS (matrix: α-CHCA): *m/z* = 2822 [M]^+^.

### 4.4. Co-Assembly of BODIPY-β-Annulus Peptides and β-Annulus Peptides in Solutions Containing PEG

Buffer containing 5, 10, 15, and 20 wt% PEG_2000_ were prepared by mixing 10 mM Tris-HCl buffer (pH 7.0) with PEG_2000_. Aqueous solution of *β*-annulus peptide (0~200 μM) and BODIPY-*β*-annulus peptide (0.1 μM) was lyophilized. A total of 10 mM Tris-HCl buffer (pH 7.0) or buffer containing 5, 10, 15, and 20 wt% PEG_2000_ was added to the dried *β*-annulus and BODIPY-*β*-annulus peptide powder. The mixture was sonicated for 10 min and incubated for 30 min at 25 °C before FCS analysis.

### 4.5. Fluorescence Correlation Spectroscopy (FCS)

A sample solution (20 μL) was put on a well and measured at 25 °C. Analyses were performed Hamamatsu Photonics Control FCS software. The diffusion time (*τ*) and ratio (*y*) of fast and slow components were obtained by curve-fitting of autocorrelation function *G*(*t*) obtained from FCS measurements according to (Equations (1) and (2)):

(1) $$G\left(t\right)=1+\frac{1}{N}\times \left(\frac{y}{\left(1+\frac{t}{\tau }\right)\sqrt{1+\frac{1}{k^{2}}\cdot \frac{t}{\tau }}}\right)\times \left(\frac{1+Fexp\left(\frac{t}{\tau_{trip}}\right)}{1-F}\right)$$

(2) $$G\left(t\right)=1+\frac{1}{N}\times \left(\frac{y_{1}}{\left(1+\frac{t}{\tau_{1}}\right)\sqrt{1+\frac{1}{k^{2}}\cdot \frac{t}{\tau_{1}}}}+\frac{y_{2}}{\left(1+\frac{t}{\tau_{2}}\right)\sqrt{1+\frac{1}{k^{2}}\cdot \frac{t}{\tau_{2}}}}\right)\times \left(\frac{1+Fexp\left(\frac{t}{\tau_{trip}}\right)}{1-F}\right)$$

where *τ* is the diffusion time of BODIPY-*β*-annulus peptides in the detection area, *N* is the average number of BODIPY-*β*-annulus peptides in the detection area, *k* is the structural parameter. Hydrodynamic radius (*r*) of BODIPY-*β*-annulus peptide was calculated by (Equations (3) and (4)):

(3) $$\tau =\frac{\omega^{2}}{4D}$$

(4) $$D=\frac{k_{B}T}{6\pi \eta r}$$

where ω is the radius of the detection area, *T* is the absolute temperature, *k_B_* is the Boltzman constant, *η* is the viscosity of the solvent. The value of ω was evaluated by reference measurement using Alexa 488 (*D* = 414 μm^2^/s). Diffusion time of Alexa 488 measured by us was 0.0335 ms. The viscosity of the PEG_2000_ aqueous solution were estimated by measuring diffusion time of Alexa 488 (0.01 μM) in 10 mM Tris-HCl buffer (pH 7.0) containing 0~20 wt% PEG_2000_ (Table S1).

To calculate the dissociation constant *K_d_* of BODIPY-labeled artificial viral capsids, the following Equation (5) (Hill’s equation) was used:

(5) $$\log\left(\frac{\Delta Y}{Y_{max}-\Delta Y}\right)=n\;\log\frac{1}{K_{d}}+n\;\log\left[\beta Annulus\right]$$

where *Y_max_* is the maximum ratio of the slow component, Δ*Y* is the difference between *Y_max_* and the ratio of the slow component at the concentration, *n* is the Hill coefficient.

## Figures and Tables

**Table 1:** *K_d_* and Δ*G* calculated for the formation of artificial virus capsids using the ratio of the slow component.

Solvent	*K_d_*/μM	Δ*G*/kJ mol^−1^
10 mM Tris-HCl buffer (pH 7.0)	45.2 ± 10.6	−24.8 ± 0.739
5 wt% PEG_2000_	14.4 ± 7.81	−27.6 ± 0.899
10 wt% PEG_2000_	6.69 ± 3.25	−29.5 ± 0.952
15 wt% PEG_2000_	4.24 ± 3.22	−30.7 ± 1.27
20 wt% PEG_2000_	5.66 ± 2.15	−29.9 ± 0.710

## Data Availability

Not applicable.

## Supplementary Materials

Supplementary materials can be found at https://www.mdpi.com/article/10.3390/ijms22094754/s1.
