# Supplementary material for: Fluorescence Correlation Spectroscopy Analysis of Effect of Molecular Crowding on Self-Assembly of β-Annulus Peptide into Artificial Viral Capsid
Source: Int J Mol Sci. 2021 Apr 30;22(9):4754. doi: 10.3390/ijms22094754 (PMC8125178; doi:10.3390/ijms22094754)
Supplement: Supplementary file 1 [file ijms-22-04754-s001.zip › ijms-1171155-supplementary.pdf]

Supplementary Materials

Fluorescence Correlation Spectroscopy Analysis of Effect of Molecular Crowding on Self-Assembly of *β*-Annulus Peptide into Artificial Viral Capsid

Risako Kobayashi,[a] Hiroshi Inaba,[ab] and Kazunori Matsuura *[ab]

[a] Department of Chemistry and Biotechnology, Graduate School of Engineering, Tottori University, Koyama-Minami 4-101, Tottori 680-8552, Japan

[b] Centre for Research on Green Sustainable Chemistry, Tottori University, Koyama-Minami 4-101, Tottori 680-8552, Japan

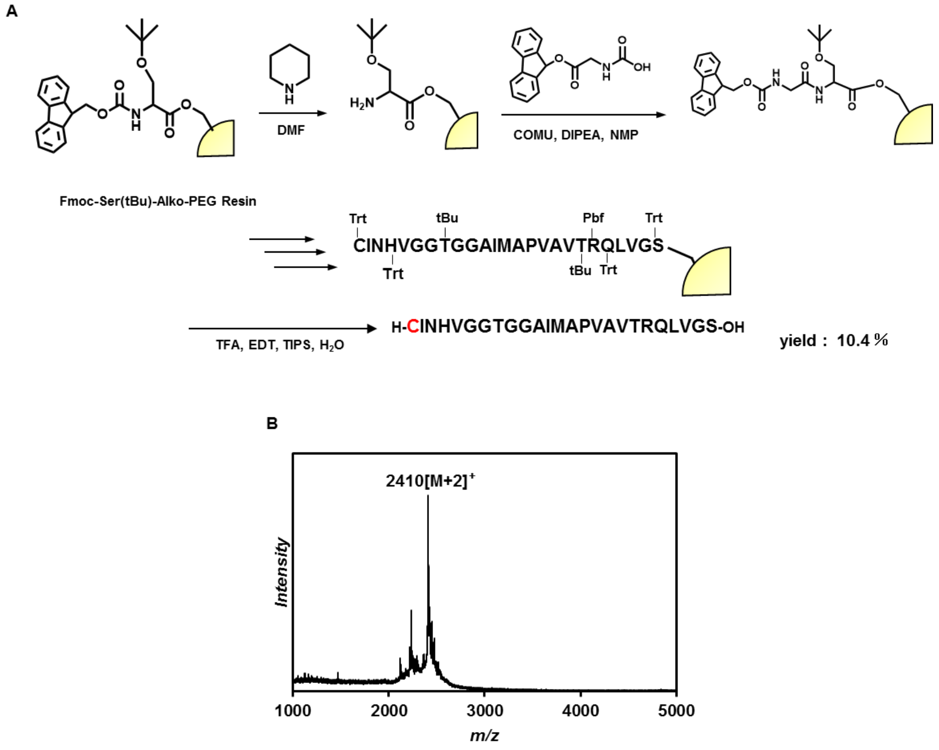

Figure S1. Synthesis of Cys-*β*-annulus peptide by solid-phase Fmoc-chemistry (A) and MALDI-TOF-MS (B).

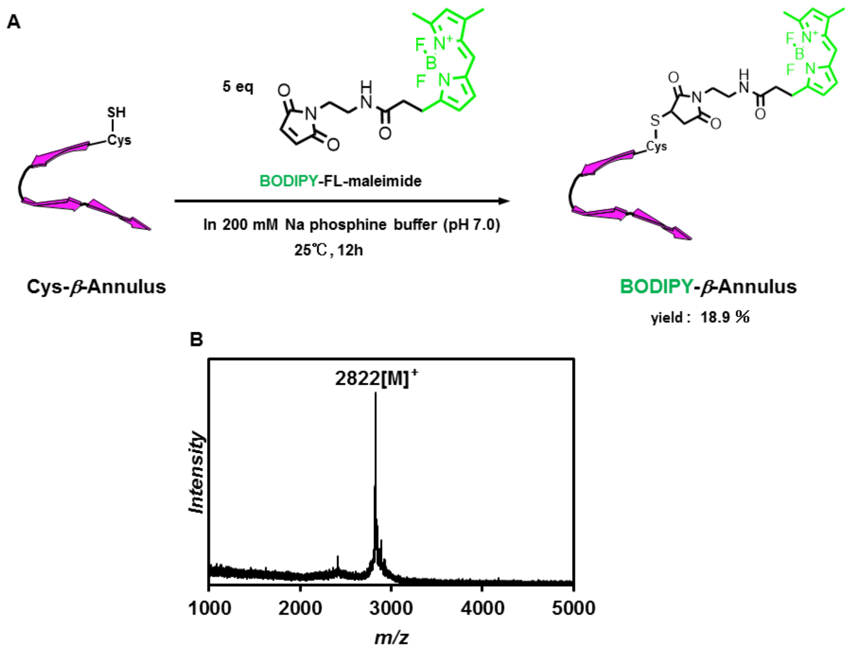

Figure S2. Synthesis of BODIPY-*β*-annulus peptide (A) and MALDI-TOF-MS (B).

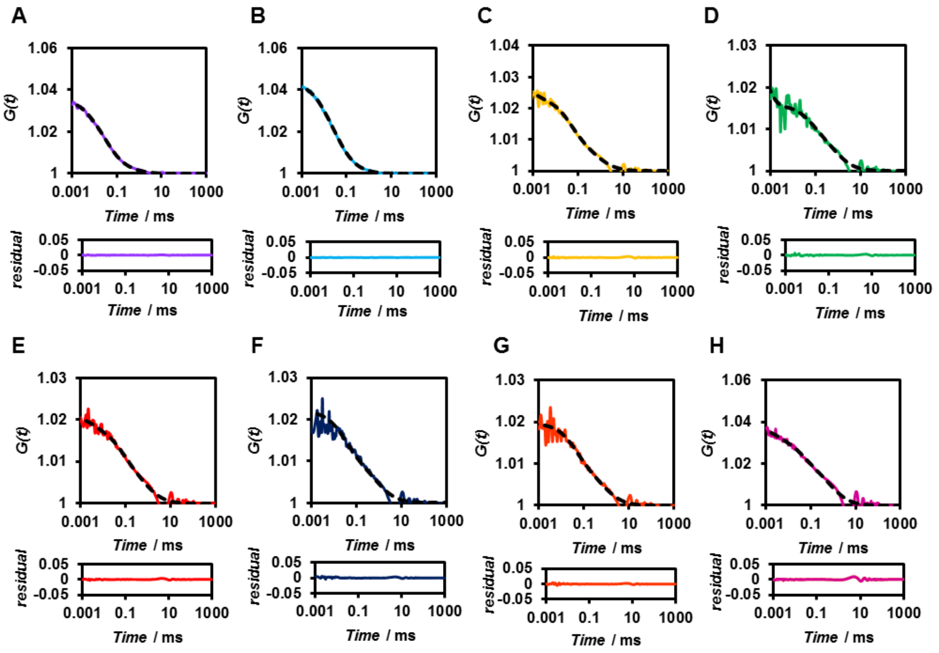

Figure S3. Measured (solid) and fitted (dot) autocorrelation curves for 0.1 μM BODIPY-*β*-annulus (A) and mixture of 0.1 μM BODIPY-*β*-annulus and 10 (B), 25 (C), 50 (D), 75 (E), 100 (F), 150 (G), 200 μM (H) *β*-annulus peptide measured by fluorescence correlation spectroscopy in 10mM Tris-HCl buffer (pH 7.0). The lower graph shows residual plot.

Table S1. Viscosity of solution in the presence of PEG.

Solvent	Diffusion time of Alexa 488 / ms	Viscosity / mPa*s
10 mM Tris-HCl buffer(pH 7.0)	0.0335	0.893
5 wt% PEG_{2000}	0.0514	1.34
10 wt% PEG_{2000}	0.0726	1.89
15 wt% PEG_{2000}	0.143	3.73
20 wt% PEG_{2000}	0.163	4.25

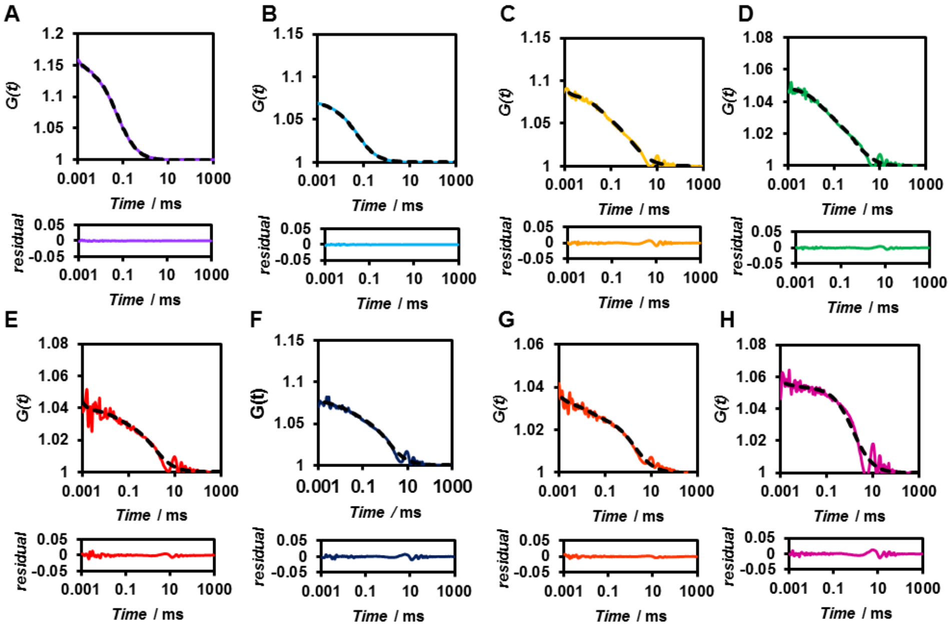

Figure S4. Measured (solid) and fitted (dot) autocorrelation curves for 0.1 μM BODIPY-*β*-annulus (A) and mixture of 0.1 μM BODIPY-*β*-annulus and 10 (B), 25 (C), 50 (D), 75 (E), 100 (F), 150 (G), 200 μM (H) *β*-annulus peptide in 5 wt% PEG_{2000} solution measured by fluorescence correlation spectroscopy. The lower graph shows residual plot.

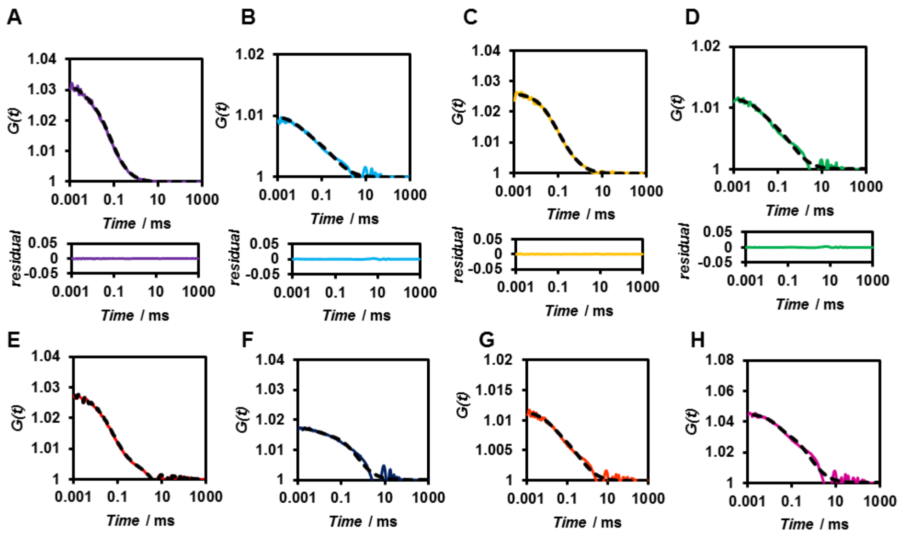

Figure S5. Measured (solid) and fitted (dot) autocorrelation curves for 0.1 μM BODIPY-*β*-annulus (A) and mixture of 0.1 μM BODIPY-*β*-annulus and 10 (B), 25 (C), 50 (D), 75 (E), 100 (F), 150 (G), 200 μM (H) *β*-annulus peptide in 10 wt% PEG_{2000} solution measured by fluorescence correlation spectroscopy. The lower graph shows residual plot.

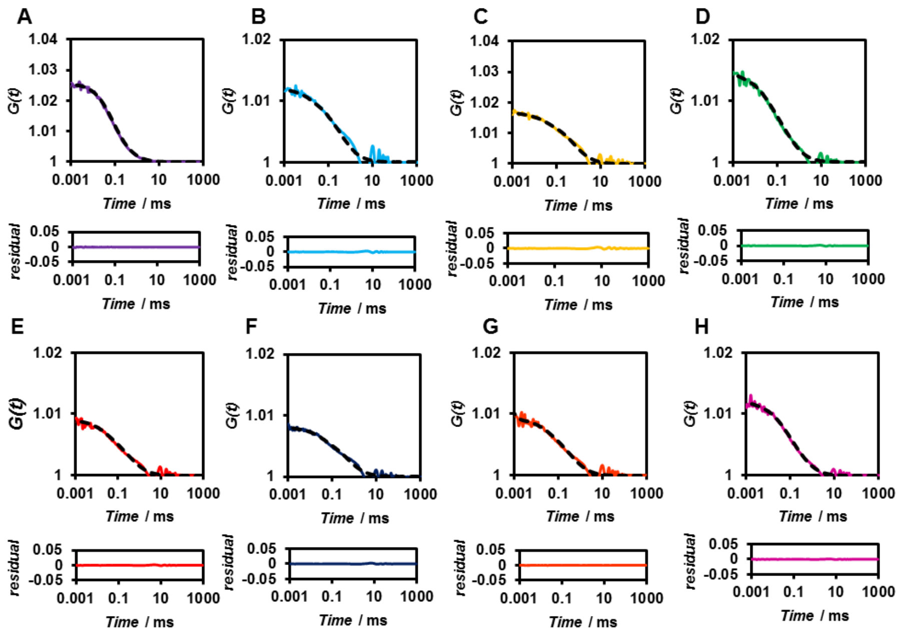

Figure S6. Measured (solid) and fitted (dot) autocorrelation curves for 0.1 μM BODIPY-*β*-annulus (A) and mixture of 0.1 μM BODIPY-*β*-annulus and 10 (B), 25 (C), 50 (D), 75 (E), 100 (F), 150 (G), 200 μM (H) *β*-annulus peptide in 15 wt% PEG_{2000} solution measured by fluorescence correlation spectroscopy. The lower graph shows residual plot.

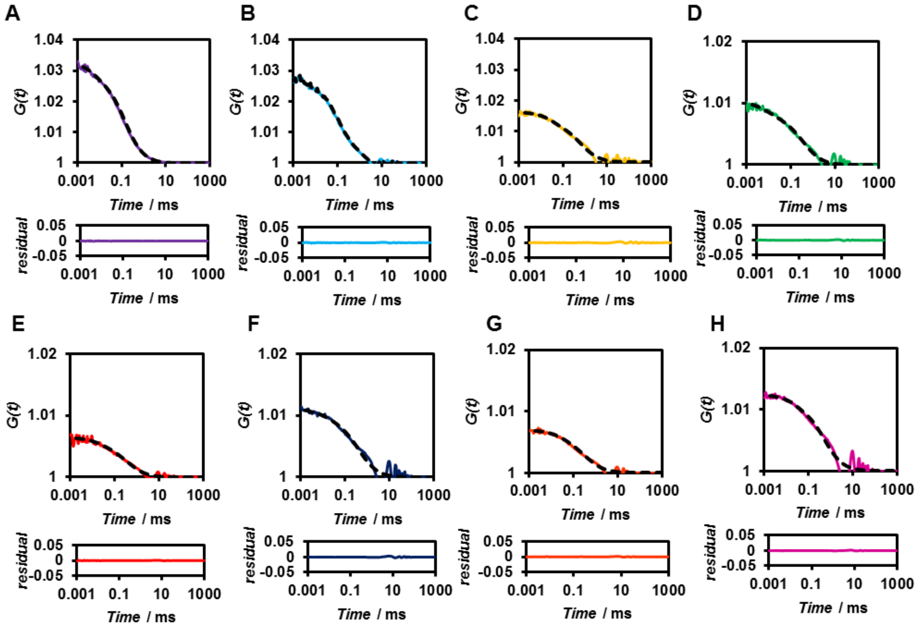

Figure S7. Measured (solid) and fitted (dot) autocorrelation curves for 0.1 μM BODIPY-β-annulus (A) and mixture of 0.1 μM BODIPY-β-annulus and 10 (B), 25 (C), 50 (D), 75 (E), 100 (F), 150 (G), 200 μM (H) β-annulus peptide in 20 wt% PEG_{2000} measured by fluorescence correlation spectroscopy. The lower graph shows residual plot.

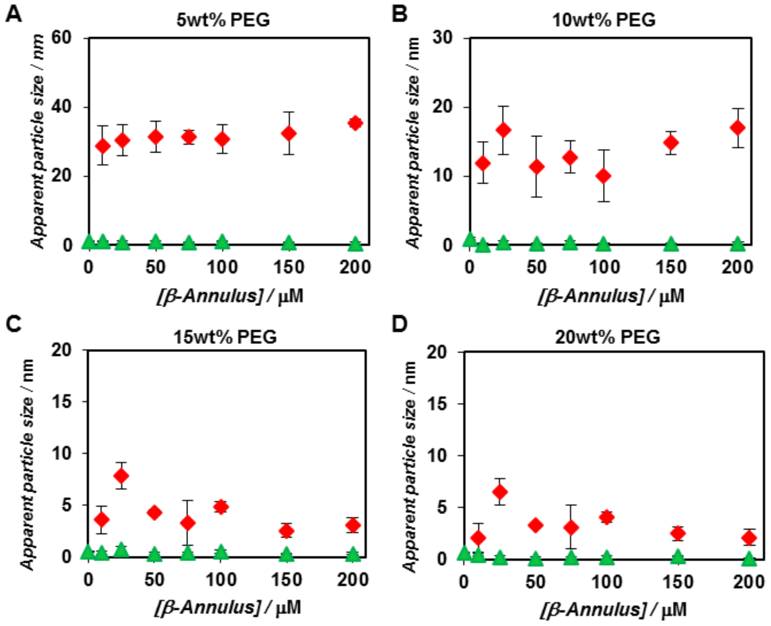

Figure S8. *β*-annulus peptides concentration dependence of the apparent diameter (A ~ D) and of the fast component (green) and the slow component (red) determined by FCS curve fitting (*N* = 3) at constant 0.1 μM BODIPY-*β*-annulus peptide under 5 wt% (A), 10wt% (B), 15wt% (C), 20wt% (D) PEG_{2000} at 25 ℃.

Table S2. Effect of PEG on BODIPY-*β*-annulus peptide.

	Diffusion time of 0.1 μM BODIPY-*β*-annulus peptide / ms	Apparent diameter / nm
10 mM Tris-HCl buffer (pH 7.0)	0.0301	1.00
5 wt% PEG_{2000}	0.0431	1.12
10 wt% PEG_{2000}	0.0631	0.937
15 wt% PEG_{2000}	0.0776	0.541
20 wt% PEG_{2000}	0.107	0.643
